# Disease state associated with chronic toe lesions in hellbenders may alter anti-chytrid skin defenses

**DOI:** 10.1038/s41598-023-28334-4

**Published:** 2023-02-03

**Authors:** Rebecca H. Hardman, Laura K. Reinert, Kelly J. Irwin, Kendall Oziminski, Louise Rollins-Smith, Debra L. Miller

**Affiliations:** 1 Department of Biomedical and Diagnostic Sciences, University of Tennessee College of Veterinary Medicine, Knoxville, TN 37996 USA; 2 Department of Pathology, Microbiology and Immunology, Vanderbilt University School of Medicine, Nashville, TN 37232 USA; 3 Arkansas Game and Fish Commission, Benton, AR 72015 USA; 4 Florida Fish and Wildlife Conservation Commission, Fish and Wildlife Research Institute, St. Petersburg, FL 33701 USA; 5 School of Natural of Resources, University of Tennessee, Knoxville, Tennessee 37996 USA

**Keywords:** Peptides, Conservation biology, Diseases, Herpetology, Fungal pathogenesis

## Abstract

Hellbenders (*Cryptobranchus alleganiensis*) are large, aquatic salamanders from the eastern United States. Both subspecies, eastern and Ozark hellbenders, have experienced declines resulting in federal listing of Ozark hellbenders. The globally distributed chytrid fungus, *Batrachochytrium dendrobatidis (Bd)* has been detected in both subspecies, and *Batrachochytrium salamandrivorans* (*Bsal*) poses a new threat if introduced into North America. Ozark hellbenders also suffer a high prevalence of toe lesions of unknown etiology, with changes in host immunocompetence hypothesized to contribute. Antimicrobial peptides (AMPs) secreted from dermal granular glands may play a role in hellbender health. We collected skin secretions from free-ranging hellbenders and enriched them for small cationic peptides used for growth inhibition assays against *Bd* and *Bsal*. Generalized linear mixed models revealed the presence of active toe lesions as the strongest and only significant predictor of decreased *Bd* inhibition by skin peptides. We also found skin secretions were more inhibitory of *Bsal* than *Bd*. MALDI-TOF mass spectrometry revealed candidate peptides responsible for anti-chytrid activity. Results support the hypothesis that hellbender skin secretions are important for innate immunity against chytrid pathogens, and decreased production or release of skin peptides may be linked to other sub-lethal effects of disease associated with toe lesions.

## Introduction

Amphibians are the most rapidly declining vertebrate group with over 41% listed as threatened or endangered by the IUCN Red List^1^. Disease is the leading cause of amphibian extinctions across the globe and remains a major threat to extant species. Most of these disease-driven declines can be attributed to novel introduction of the chytrid fungus (*Batrachochytrium dendrobatidis*, *Bd*). The global pandemic lineage (GPL) of *Bd* is endemic to Asia^2^ and is estimated to be responsible for 90 extinctions in less than three decades^3^. This has given much deserved attention to highly susceptible frog species such as the Panamanian golden frog (*Atelopus zeteki*)^4^. However, other species may not be as susceptible with subsequently less obvious disease or declines. This includes many salamander species that have experienced noticeable declines in conjunction with arrival of *Bd* but without large outbreaks^5^. More recently, an emerging chytrid pathogen *B. salamandrivorans* (*Bsal*), has been introduced into Europe causing mass mortalities of the once common fire salamander (*Salamandra salamandra*)^6^. Unlike *Bd*, *Bsal* appears to affect salamanders more than frogs^7^. Ranavirus is another important amphibian pathogen responsible for mass mortality events most notably in temperate regions of North America^8^ and Europe^9^. While ranaviruses have not caused extinctions like *Bd,* they have caused declines in once common species such as the common frog, *Rana temporaria*^10^ and have a more diverse host range^11^. This highlights the need for conservation efforts and baseline health information for all amphibians, including salamanders.

The hellbender (*Cryptobranchus alleganiensis*) is a large fully aquatic salamander found in streams and rivers within the eastern and midwestern United States. Hellbenders are important both genetically and ecologically. They represent one of only three species from the giant salamander family, Cryptobranchidae, and are the largest salamander of North America. Adults can grow to over 60 cm in total length and 1 kg mass^12^. They are also important long-lived top stream predators. They feed on a variety of crayfish and fish species as adults and have a lifespan of 30 + years^12^. Two subspecies, the eastern hellbender (*C. a. alleganiensis*) and Ozark hellbender (*C. a. bishopi*), have both experienced considerable range retraction and decreased population densities over at least the past 25 years^13,14^. Ozark hellbenders are restricted to rivers of the Ozark highlands of Missouri and Arkansas and because of rapid declines were listed as endangered by the US Fish and Wildlife Service (USFWS) in 2011^15^. The eastern hellbender is estimated to have 77.9% of historical populations extirpated or in decline, but because the remaining 22.1% are considered relatively stable, it is not currently considered as a candidate for listing^16^.

Habitat degradation and sedimentation is associated with the majority of observed declines^17,18^ but an immediate mechanism for these declines is unknown. Chytridiomycosis (disease due to chytrid infection) has been hypothesized to contribute, but previous studies have been inconclusive in support of this. *Bd* has been detected in hellbenders as early as 1969^19^, and in the past decade, it has been found in both healthy and unhealthy hellbender populations ranging from 0 to 33% prevalence^20–24^. Chytridiomycosis has only been documented in captive and captive-raised populations with associated mortalities as high as 100%^25,26^. Like *Bd*, ranavirus has largely unknown effects in wild hellbenders, having only been detected subclinically^20,22^, however, infection is significantly associated with a decrease in body condition^20^ suggesting sublethal effects. One study showed that at least one strain of ranavirus can cause mortality in juvenile hellbenders^27^. In the closely related Chinese giant salamander (*Andrias davidianus*), another strain has devastated captive colonies causing severe hemorrhage, skin ulcerations, and mortality^28^.

These above known amphibian pathogens, along with many other unknown infectious agents, have the potential to impact hellbender populations given the right conditions. Unfortunately, over 90% of Ozark hellbenders in Arkansas have a form of distal limb lesion characterized by ulceration and digital necrosis^29^. Although there is no definitive etiology for these chronic and progressive lesions, *Bd* infection is associated with increased severity^29^. A process involving decreased host immunity and decreased wound healing is hypothesized to contribute to lesion formation^29,30^. More recently, similar toe lesions have been discovered in eastern hellbenders^20,31^, but data are limited as to whether they arise from a similar pathology as those seen in Ozark hellbenders.

The skin is an organ particularly important for amphibians because it is a major site for electrolyte exchange and homeostasis ^32^. This may be especially true for hellbenders which rely almost exclusively on cutaneous respiration after larvae resorb gills around one year of age^33^. Therefore, knowledge of the dynamics of skin immune function in hellbenders may be valuable for assessing both individual and population health. Granular (poison) glands are one group of specialized serous glands on amphibian skin that produce a variety of products including small peptides with antimicrobial activity called host defense peptides or antimicrobial peptides (AMPs)^34^. AMPs are critical components of innate immunity in all vertebrates and represent hundreds of varied peptides produced in many animal tissues with a large spectrum of activity^35^. These 10–50 residue peptides are cationic, amphipathic, and can have direct antimicrobial activity via membrane disruption of bacteria, viral envelopes, fungi, and protozoa^35^. This non-specific targeting of cellular membranes makes AMPs an important tool for pathogen deactivation and killing.

More than 2000 amphibian AMPs have been described^36^. AMPs isolated from granular glands of frogs have demonstrated growth inhibition of *Bd* and other known amphibian pathogens^37–39^. AMPs are likely also involved in other aspects of skin function. For instance, skin AMPs from various frog species can promote healing when applied to wounds on mouse skin^40,41^. Differences in AMP secretion have been associated with interspecific^37^ and regional intraspecific^42^ variation in resistance to *Bd*. Further, variation in AMP secretion can be found within individuals before and after a stress event^43^ revealing how AMPs may be an important avenue through which environmental stressors can impact individual and population health.

Salamander AMPs are hypothesized as one factor responsible for increased resistance of salamanders to *Bd* in comparison to many anuran species^39^. However, despite the impressive amount of information available on AMPs of anurans, there are strikingly few studies on those of salamanders. Skin peptides have been harvested from only a few salamander species and show varied inhibition of several pathogens such as *Bd*, *Bsal*, *Escherichia coli*, and at least one FV3-like ranavirus strain^44–49^. Hellbenders are colloquially known as snot otters because of the copious volume of skin secretions they can produce while being handled, and these secretions could contain an important component of hellbender skin immunity. To date only one published study has evaluated AMPs from hellbender skin^44^, and only one AMP has been characterized for the entire Cryptobranchid family, from Chinese giant salamanders^50^. Given that *Bd* and *Bsal* both pose a conservation risk to hellbenders, and various skin lesions are prevalent within populations, we investigated whether hellbenders had skin peptides effective against both chytrid fungi. We further evaluated how that activity might change across regions of varying population status and individuals of varying infection and health status.

## Results

### *Bd* growth inhibition assays (GIAs)

**Figure 1:**
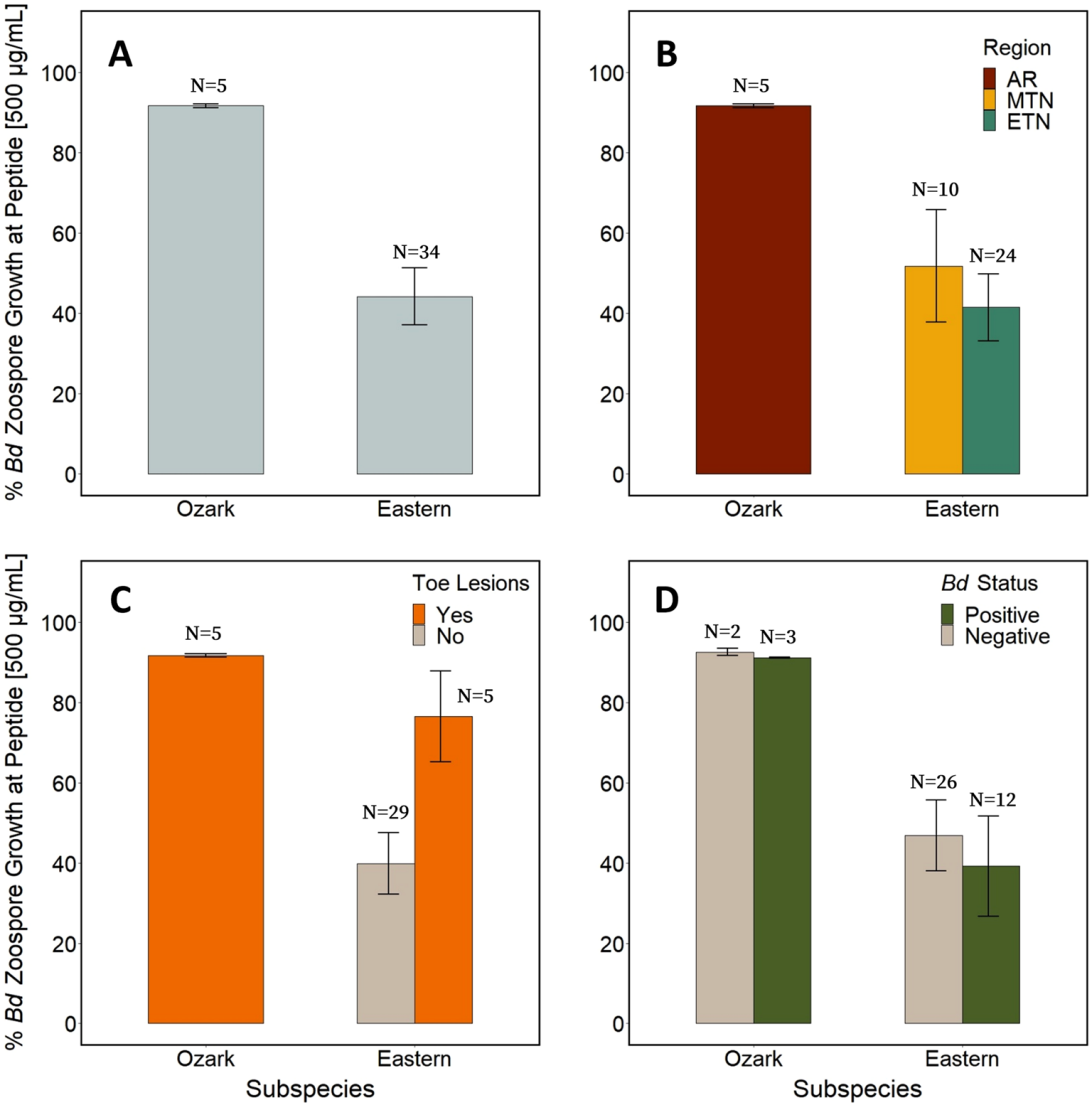
Bar graphs of average zoospore growth at 500 µg/mL. Graphs represent GIA results of % *Batrachochytrium dendrobatidis* (*Bd*) zoospore growth at 500 µg/mL hellbender skin peptide concentrations compared to positive controls based on different group parameters. Note that a decrease in zoospore growth denotes an increase in zoospore inhibition. Standard error bars present. Variation in zoospore growth grouped by subspecies (**A**), capture region within subspecies (**B**), presence/absence of toe lesions at time of collection (**C**), and *Bd* status via qPCR from skin swabs taken at time of collection (**D**).

**Figure 2:**
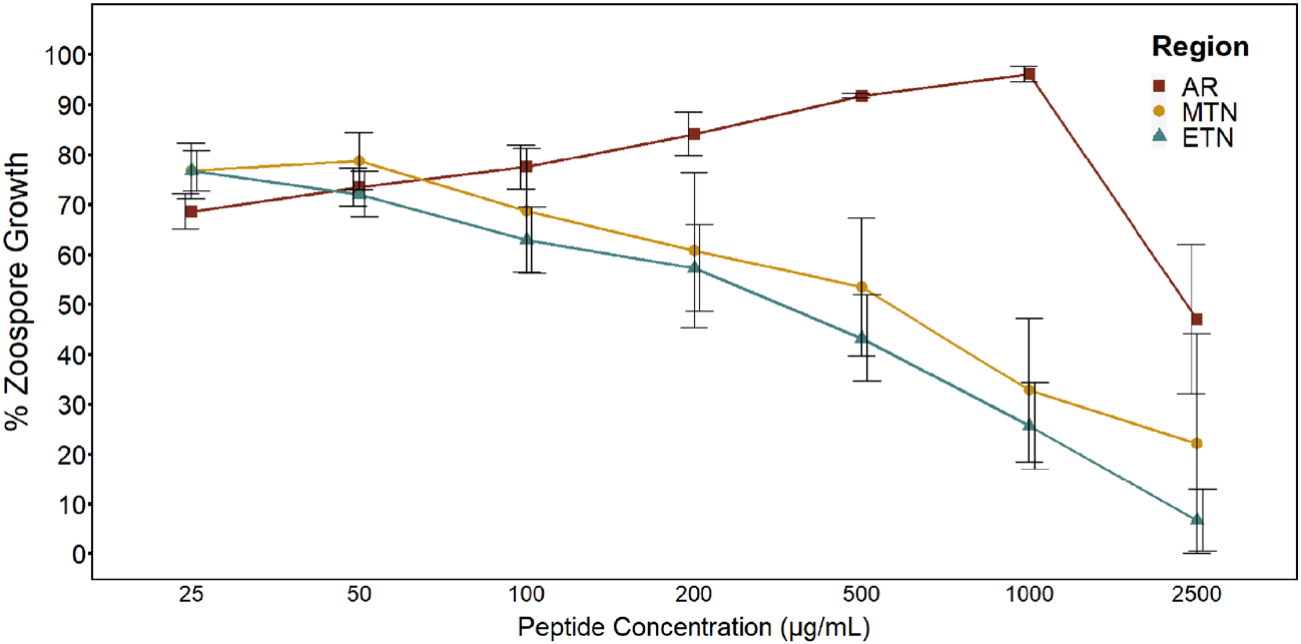
*Bd* inhibition curves of hellbender skin peptides grouped by region. Line graphs show inhibition curves with standard error bars of % *Bd* zoospore growth based on positive controls against increasing concentrations of skin peptides collected from both hellbender subspecies: Ozark hellbenders are from Arkansas (AR; red), and eastern hellbenders from Middle Tennessee (MTN; yellow), and East TN (ETN; teal). Anti-*Bd* activity of peptides from all individuals were tested up to 1000 µg/mL (n = 39; 5 AR, 10 MTN, 24 ETN) with a subset tested out to 2500 µg/mL (n = 16; 5 AR, 5 MTN, 6 ETN).

We performed growth inhibition assays (GIAs) against *Bd* using skin peptides collected from 39 hellbenders [5 from Ozark hellbender populations of Arkansas (AR), 10 from eastern hellbenders of Middle Tennessee (MTN), and 24 from eastern hellbenders of East Tennessee (ETN)]. Of these 39 individuals, 10 had active toe lesions (5/5 Ozark, 5/34 eastern), 15 were *Bd* positive (3/5 Ozark, 12/34 eastern), and 4 were ranavirus positive (0/5 Ozark, 4/34 eastern). Overall, skin secretions from AR Ozark hellbenders had limited inhibition of *Bd* in comparison with eastern hellbenders (Fig. 1A). Specifically, in response to secretions from AR hellbenders, the average % zoospore growth (compared to controls) at peptide concentrations of 500 µg/mL was 91.7% compared with 53.4% for MTN and 43.1% for ETN (Fig. 1B). AR peptides did not exhibit a typical inhibition curve by having an increase in zoospore growth from 25 to 1000 µg/ mL. Only after this threshold of 1000 µg/ mL did *Bd* growth begin to decrease with increased peptide concentrations (Fig. 2). We performed additional GIAs with an added concentration of 2500 µg/ mL on a subset of 16 animals (AR = 5, MTN = 5, ETN = 6) and found almost all peptides from eastern hellbenders to completely inhibit zoospores at a concentration of 2500 µg/mL (growth at 22.1% for MTN, 6.6% for ETN), whereas those from Ozark hellbenders on average inhibited only half of zoospore growth (growth at 46.9%) (Fig. 2).

AIC_c_ evaluation of GLMMs of binomial inhibition (> 50% vs. < 50% zoospore growth at 500 µg/mL) revealed three top models with Δ AIC_c_ < 2: Toe Lesion-Only, Toe Lesion + Mass, and Toe Lesion + *Bd* (Table 1). We model-averaged the three variables present in top models (Toe Lesion, Mass, and *Bd*) and found Toe Lesion to be significantly positively correlated with *Bd* zoospore growth (β = 3.45; C.I. 0.97, 5.92; Table 2). Exposure of *Bd* zoospores to peptides of hellbenders with no toe lesions had on average 39.9% *Bd* zoospore growth at 500 µg/mL in comparison to those exposed to peptides of hellbenders with toe lesions (85.0% zoospore growth) (Fig. 1C). Peptides from hellbenders positive for *Bd* on average had weaker inhibition of *Bd* zoospores (57.8% growth) compared to peptides from negative individuals (53.2% growth), but this was not a significant effect in our GLMMs (β = 0.07; C.I. − 1.48, 1.63) (Table 2, Fig. 1D). There was no significant effect of mass on peptide activity (β = − 0.54; C.I. − 1.6, 0.52; Table 2).

**Table 1:** List of Generalized Linear Mixed Models (GLMM) (binomial distribution) included in AICc analysis in order of smallest to largest ΔAICc.

Model	Fixed Effects	Random effect	K	AICc	ΔAICc	AICcWt
**Mod2**	**Toe**	**Year**	**3**	**46.35**	**0**	**0.36**
**Mod6**	**Toe + Mass**	**Year**	**4**	**47.72**	**1.38**	**0.18**
**Mod3**	**Toe + *Bd***	**Year**	**4**	**48.18**	**1.84**	**0.14**
Mod4	Toe + Region	Year	4	48.83	2.49	0.1
Mod5	Toe + Mass + Region	Year	5	49.44	3.1	0.08
Mod8	Toe + Mass + *Bd*	Year	5	49.83	3.48	0.06
Mod7	Toe + *Bd* + Region	Year	5	50.85	4.5	0.04
Mod1	Mass + Region + Toe + *Bd*	Year	6	52.04	5.69	0.02
Mod9	Region	Year	3	54.83	8.49	0.01
Mod11	*Bd* + Region	Year	4	56.05	9.7	0
Mod15	Mass + Region	Year	4	56.54	10.19	0
Mod10	Mass + Region	Year	4	56.54	10.19	0
Mod17	–	Year	2	57.27	10.92	0
Mod12	Mass + *Bd* + Region	Year	5	58.21	11.87	0
Mod13	Mass	Year	3	58.24	11.89	0
Mod16	*Bd*	Year	3	58.67	12.32	0
Mod14	Mass + *Bd*	Year	4	59.37	13.03	0

Models were based on hellbender skin peptide inhibition of *Bd* zoospores at 500 µg/ mL compared to positive controls categorized into weak (51–100% zoospore growth) or strong (0–50% zoospore growth) inhibition. Top models of ΔAICc < 2.0 are noted in bold and were used for subsequent variable model averaging.

**Table 2:** Fixed effects variables in top models from GLMM.

Variable	β Coeff	S.E	95% C.I
**Toe**	**3.45**	**1.26**	**0.97, 5.92**
Mass	− 0.54	0.54	− 1.6, 0.52
*Bd*	0.07	0.79	− 1.48, 1.63

(ΔAICc < 2) listed with model-averaged results. Significant effects (based on 95% C.I.) noted in bold.

For evaluation of effects of ranavirus infection on skin peptide activity we compared peptides from six individuals collected from the same site in ETN on the same day. Two individuals were ranavirus-positive, two were co-infected with ranavirus and *Bd*, and two were negative for both pathogens. Of this group, peptides from completely negative individuals and coinfected individuals displayed full inhibition of *Bd* zoospores at 500 µg/mL with (0.0% and 0.7% zoospore growth, respectively) in comparison to ranavirus-positive individuals (66.7% growth).

### *Bsal* growth inhibition assays (GIAs)

**Figure 3:**
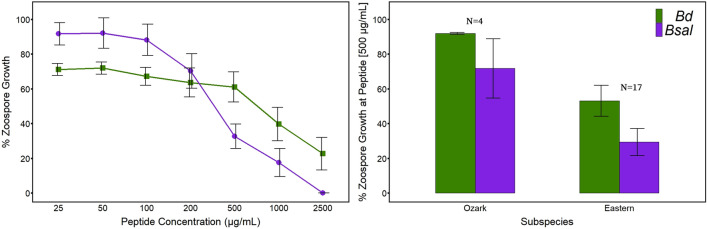
Differential inhibition of two chytrid species. *Batrachochytrium dendrobatidis* (*Bd*; green) and *B. salamandrivorans* (*Bsal*; purple), show different inhibition profiles when challenged against varying hellbender skin peptides concentrations. Left shows curves with standard error bars of % zoospore growth in comparison to positive controls against increasing peptide concentrations of 25 to 2500 µg/mL. Right shows bar graphs with standard error bars representing % zoospore growth at 500 µg/mL peptide concentrations, separated by subspecies of Ozark hellbenders of Arkansas and eastern hellbenders of Middle and East Tennessee. Growth inhibition assays for *Bd* and *Bsal* comparisons were performed from the same peptide samples (n = 21, 4 Ozark, 17 eastern).

In addition to *Bd* GIAs, we performed *Bsal* GIAs from 21 of the 39 peptide samples (4 from AR, 7 from MTN, and 10 from ETN; Fig. 3). Paired samples Wilcoxon Rank Sum test showed hellbender peptides significantly inhibited *Bsal* better than *Bd* with an average drop in zoospore growth at 500 µg/mL from 50.3% to 36.0% for *Bd* and *Bsal*, respectively (*P* = 0.034). Peptides from AR showed increased, but still limited, inhibition of *Bsal* zoospores (71.8% growth) compared to *Bd* (91.7% growth) and remained weaker in comparison to those collected from MTN and ETN regions. Peptides from MTN had the greatest differential effects on *Bd* versus *Bsal* growth (53.4% and 17.8%, respectively), with little change between inhibition of chytrid species for ETN (43.1% and 34.5% *Bd* and *Bsal* growth, respectively; Fig. 3). Only 3/21 (14.3%) of individuals had peptides that displayed weaker inhibition of *Bsal* compared to *Bd* and all were from ETN.

### MALDI-TOF

**Figure 4:**
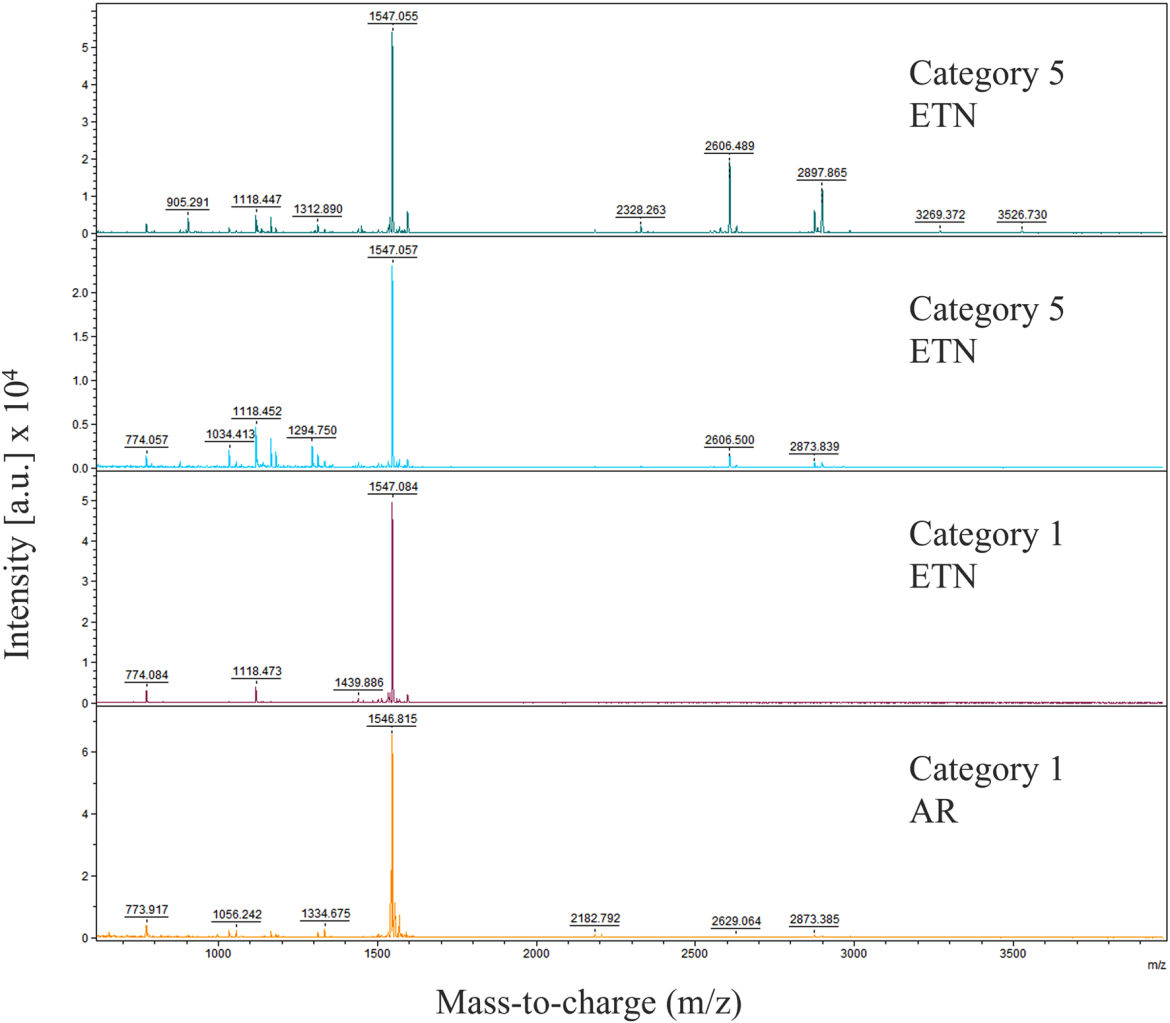
MALDI-TOF profiles from 1000 to 4000 Da of four skin peptide samples collected passively from hellbenders. Comparison can be seen between the most inhibitory samples of *Batrachochytrium dendrobatids* at 500 µg/mL peptide concentration (*Bd* zoospore growth < 10%; Category 5), and the least inhibitory samples (*Bd* zoospore growth > 90%; Category 1). Top two profile are from individuals of eastern hellbenders from East Tennessee (ETN) that exhibited complete inhibition of *Bd* zoospores (Category 1). The bottom two profiles include an eastern hellbender from ETN and an Ozark hellbender from AR that exhibited very little *Bd* zoospore inhibition (Category 5). While profiles showed considerable variation, all samples evaluated included strong peaks around m/z 1547. Note also that the two samples of Category 5 have peaks of around m/z 1122 as well as either peaks around m/z 2879 or m/z 2896; these three peptide masses were considered indicative of strong inhibition based on indicator species analysis.

We performed matrix-assisted laser desorption time-of-flight (MALDI-TOF) mass spectrometry on enriched peptides from 19 individuals. We created a list of the most frequent peptides with largest isotope cluster areas and found m/z weights of 1530–1560 to be the most abundant across all samples with peptides of m/z 1547 to have the strongest relative intensity and isotope cluster area (Table 3). Indicator species analysis revealed peaks of five molecular weights that best distinguished strongest inhibition of *Bd* zoospores (Category 5) from weakest inhibition (Category 1) (Table 4). These candidate peptides were found in both higher prevalence and relative intensity in Category 5 versus Category 1 samples (Fig. 4). No peptides identified were exclusive to any one region.

**Table 3:** List of peptides (identified by m/z) with largest average cluster area across all skin peptides collected from 19 hellbenders individually analyzed via MALDI-TOF MS.

Peptide	Average cluster area by inhibition category (1–5)					
Mass (m/z)	Total	**5** (n = 6)	**4** (n = 2)	**3** (n = 4)	**2** (n = 6)	**1** (n = 4)
1547	448.4	533.9	722.0	53.7	504.5	493.8
1530	337.7	493.8	378.9	168.5	239.5	399.2
1529	327.2	491.8	311.0	163.7	197.4	446.4
1546	263.6	531.6	0.0	371.7	0.0	280.9
1506	196.3	230.2	265.7	65.6	159.4	297.0
1560	181.3	289.5	531.1	134.5	44.6	96.2
1543	161.3	98.2	335.3	19.2	57.8	466.2
1165	152.7	251.9	244.1	50.2	26.9	249.3
2605	139.7	323.4	393.8	31.7	0.0	54.8
1502	136.5	96.0	123.1	24.2	170.8	265.0
1439	135.0	171.4	286.2	103.4	0.0	238.8
1436	131.6	185.0	112.4	56.3	42.9	269.4
1504	124.9	183.6	133.5	0.0	57.3	258.8
1136	110.6	129.9	199.2	97.9	54.0	135.3
1122	106.8	322.0	34.0	75.7	7.9	0.0

Peptides are ranked by total average cluster area. Average cluster areas are also given for each inhibition category (1–5) based on % Bd zoospore growth of GIA results. from 1 to 5. A Category 1: 91–100% zoospore growth, Category 2: 76- 90%, Category 3: 26–75%, 4: 11–25%, and Category 5: 0–10%. Note that Category 5 had the least zoospore growth and therefore greatest zoospore inhibition.

**Table 4:** List of candidate peptides identified by m/z from MALDI-TOF MS analysis of peptides collected from passive skin secretions of 19 hellbenders and significantly associated with samples of best *Batrachochytrium dendrobatidis* (*Bd*) zoospore growth inhibition based on indicator species analysis.

Peptide Mass (m/z)	Stat.	*p*-value
2879	0.866	0.006
2896	0.866	0.013
1122	0.802	0.030
1105	0.791	0.037
2872	0.791	0.028

Inhibition categories were created by how well skin peptides at 500 µg/mL from each individual hellbender inhibited *Bd* zoospore growth from least inhibition (greatest zoospore growth) to greatest inhibition (least zoospore growth) on a scale from 1–5 where Category 1 had 91–100% *Bd* zoospore growth, Category 2: 76- 90%, Category 3: 26–75%, 4: 11–25%, and Category 5: 0–10%.

## Discussion

Hellbender skin peptides inhibited both *Bd* and *Bsal* zoospore growth. Wild hellbender populations with *Bd* infections are recorded as early as 1969; however, no confirmed cases of chytridiomycosis have been noted^19–24^. This may be due, in part, to the anti-chytrid activity provided by the peptides we identified in skin secretions. However, *Bd* infection status was not significantly associated with peptide anti-*Bd* activity. This is in contrast to other studies evaluating skin peptides in more susceptible frog populations^42,51^. Interestingly, in certain captive situations, *Bd* infection burden can increase and result in chytridiomycosis and mortality in hellbenders^25,26^. If peptides are a large contributing factor to hellbender chytrid tolerance, these sporadic mortality events suggest that certain conditions may affect the production of protective peptides.

Most known amphibian AMPs cause cellular damage to microbial organisms by reaching a threshold within the inner membrane leading to pore formation and membrane disruption^35^. The 500–1000 µg/mL concentration threshold we observed in hellbender skin peptides likely represents the point at which one or more critical AMPs have reached that critical concentration on zoospore membranes. However, at lower concentrations, these same AMPs may interact with microbial organisms in different ways and may even stimulate growth. *Bsal* zoospores growing in peptides from Ozark hellbender at low concentrations showed a marked increase in growth compared to positive controls. This may indicate that *Bsal* zoospores may be able to recognize salamander- specific skin peptides and respond by increasing growth rates as a potential adaptive mechanism for colonizing salamander skin. Other compounds may also be present in low concentrations in these peptide enriched samples that may stimulate growth below inhibitory thresholds. Perhaps this phenomenon can only be seen in peptide samples that require larger concentrations to inhibit. However, many other factors may also produce increased zoospore growth; when skin peptides do not achieve concentrations to cause damage, they may simply serve as an extra nutrient source.

We found considerable variation in skin peptide anti-chytrid activity between individuals. Ozark hellbenders (from AR) had skin peptides with the weakest inhibition for both *Bd* and *Bsal* compared to eastern hellbenders (from MTN and ETN), and Ozark populations are also experiencing the highest prevalence of disease and decline. Many other factors distinguish Ozark hellbenders from eastern hellbenders including being a genetically distinct subspecies and residing in a distinct ecoregion, the Ozark Highlands. Ozark hellbenders may also be suffering from genetic bottlenecking of small remaining populations as evidenced by their decreased immunogenetic diversity of major histocompatibility complex IIb^52^. Based on our GLMM results, presence of toe lesions is the strongest factor associated with decreased peptide activity and supports a hypothesis that individual health is more likely to be driving these peptide changes as opposed to region or phylogeny. One limitation for this conclusion is that we were only able to sample 5 Ozark individuals, all of which had toe lesions. This prevented a more powerful and unconfounded analysis of whether or not these other above-mentioned factors may in fact be playing a role.

The association between diminished in vitro peptide activity and presence of active toe lesions is still an important finding and shows evidence for skin peptide dysregulation as one potential mechanism or consequence of chronic disease observed in hellbenders. Dynamic AMP regulation in conjunction with stress, injury, or disease is supported in other host systems. For example, in human skin the AMP LL-37 can be upregulated in response to injury and accelerate wound healing and dysregulation has been proposed as a mechanism for manifestation of skin lesions in psoriasis and atopic dermatitis^53^. Evidence of differential AMP production in response to stimuli also exists in amphibian skin and can change within an individual after administration of glucocorticoids in northern leopard frogs (*Lithobates pipiens*)^43^, and African clawed frogs (*Xenopus laevis*)^54^. Further, northern leopard frogs from quiet locations exposed to mild stress of prolonged traffic noise had increased corticosteroid responses and produced fewer AMPs^42^. Hellbenders are fully aquatic, benthic organisms with their feet in full contact with rough substrate as they crawl through strong currents. If AMP dysregulation exists throughout the entire hellbender, perhaps only the toes manifest in chronic wounds as they are the regions that experience greatest trauma. The peptides recovered from the passive bath could have also been directly affected by peptides produced at the site of the toe lesions and may not represent overall physiological change. Future research should evaluate the immediate mechanisms of why changes in AMP activity is reduced in animals manifesting active toe lesions and if there is a causative link. Regardless, these results are important in revealing important immunological changes associated with observed chronic disease that may have population-level implications.

Although *Bd* infection did not have a strong association with hellbender skin peptide activity, we found a potential interaction with ranavirus coinfection at the one site we were able to sample ranavirus- positive individuals. Skin secretions from ranavirus-positive individuals had markedly decreased killing ability compared to those from ranavirus-negative individuals, but anti-chytrid activity appeared to be restored in secretions from those coinfected with *Bd*. Even though we were only able to evaluate a small subset (n = 4) of ranavirus-positive individuals from a single site, this observation highlights potentially important host–pathogen interactions. More research will be needed to determine if these observations are repeatable.

Peptides recovered from hellbender skin showed greater inhibition of *Bsal* compared to *Bd*. This was an unexpected finding because hellbender populations sampled have had exposure to *Bd* whereas they are naïve to *Bsal*^20,21^, and further, *Bsal* is thought to be more virulent to salamanders than frogs^7^. This is in line with a previous study evaluating hellbender skin peptides in which some *Bsal* and no *Bd* inhibition was observed^44^. It is likely that skin secretions in hellbenders are not “tuned” to any specific pathogens but instead provide a general defense against all potential skin pathogens. In fire salamanders, a European newt species highly susceptible to *Bsal*, skin peptide secretions were effective at killing both *Bd* and *Bsal* equally^46^, whereas mucosome washes (skin products including peptides and larger proteins) had limited killing ability and were more predictive of susceptibility. Hellbender AMPs similarly likely play an important role in skin health and chytrid tolerance, but in conjunction with many other skin factors. A more comprehensive look at hellbender skin immunity will be needed to better understand both *Bd* and *Bsal* susceptibility in this species.

Based on weight and isotope cluster, some candidate AMPs were identified for further investigation. All samples for which we performed MALDI-TOF MS had strong relative intensities for peaks of 1545–1547 m/z, and although this potential peptide group likely plays an important role in hellbender skin health, it did not explain differential inhibition of zoospores. Indicator species analysis did identify isotope clusters prominent in peptide mixtures with strong inhibition of *Bd* zoospores (categories 4 and 5) and absent in weaker inhibition categories. This association was observed regardless of capture region and was even noted among peptides from individuals of the same capture event.

Many unknowns remain about potential AMPs in hellbenders. An AMP from a Chinese giant salamander was isolated and shown to have anti-bacterial activity^50^, revealing that cryptobranchids do produce at least one AMP and there are likely more to be discovered. These peptides may not even be exclusive to granular gland secretions. Because we collected peptides passively, a large proportion may be from baseline production within cells such as keratinocytes. Furthermore, hellbenders host a rich bacterial community within the skin mucous environment. The skin microbiome is another part of amphibian skin immunity that is shown to contribute to chytrid resistance^56^, and part of this is attributed to products produced by the microbial symbionts. For instance, *Janthinobacterium lividum* is a symbiotic bacterium found in several frog species tolerant to *Bd* infection, which produces an antimicrobial compound, violacein, a compound associated with increased host survival against *Bd*^57^. Supernatants collected from other symbiotic bacteria from amphibian skin are shown to inhibit *Bd*^58^. AMPs have already been discovered to be produced by many bacterial species^59^, and it is therefore likely certain peptides on hellbender skin may also be of bacterial origin.

In summary, healthy amphibian skin is important to prevent dermal invasion of pathogens and our study shows that in vitro activity of hellbender skin peptides against both *Bd* and *Bsal* zoospores can vary greatly among individuals. Our findings that presence of toe lesions in host hellbenders were significantly associated with decreased zoospore inhibition provides an important clue into how these peptides may be critical in the role of healthy skin function. Dynamics in pathogen exposure and external stressors have the potential to change amphibian skin health and more research is needed to understand how immune parameters in the skin can change with both individual and environmental factors. These results suggest that chronic disease may have greater impacts on population health than previously recognized. Future research should focus on how environmental stressors can more subtly affect these populations, but also on how we may be able to treat or prevent these chronic processes as a management tool. We also recommend deeper investigation into these proposed antimicrobial peptides found within hellbender skin secretions to better understand which peptides drive anti-chytrid activity and which might be important in wound healing. It will also be important to evaluate activity against other invading pathogens including ranavirus to understand how this and other pathogens may threaten wild hellbenders. This study highlights that evaluation of hellbender immune health alongside dynamic threats from pathogens and environmental change will be important towards conservation of this unique stream predator with an uncertain future.

## Methods

### Stream collection

We sampled hellbenders between late May and early August from 2015 to 2017 in seven rivers from three ecoregions: Blue Ridge ecoregion of East Tennessee (ETN), Interior Plateau of Middle Tennessee (MTN), and Ozark Highlands of Arkansas (AR). We sampled for eastern hellbenders (*C. a. alleganiensis*) in MTN and ETN and Ozark hellbenders (*C. a. bishopi*) in AR. We implemented disease sampling protocols in coordination with ongoing population monitoring surveys. All sampling was conducted under observation of the USFWS permit TE66039A-0 issued to KJI and Arkansas Game and Fish Commission (AGFC) in AR and with approval from Tennessee Wildlife Resources Agency (TWRA) in MTN and ETN [Permit #’s 1525,1529 (DM) and 1877 (RHH)]. Hellbender handling and sampling were performed in accordance with relevant ethical guidelines and regulations of the University of Tennessee, TWRA, AGFC, and USFWS. In ETN and MTN, and shallower water in AR, we used standard snorkeling techniques to locate individuals. In AR, we also sampled hellbenders via artificial nest boxes and sampled deep water habitats (up to 4 m) using a hookah dive system. We captured animals encountered under cover objects and nest boxes and placed them in a clean, soft cotton or mesh bag. We kept bags submerged in the river before and after animals were processed. (University of Tennessee IACUC protocol # 2481). We changed dive gloves between animal captures.

### Field collection

Skin secretions were collected from a subset of hellbenders surveyed. We recorded total length (TL), mass, and presence of active (non-healed) toe lesions. Prior to skin secretion collection, we collected skin swabs and tail tip biopsies for *Bd* and ranavirus pathogen testing, respectively Note the ranavirus assay used is not specific to any ranavirus group but has been successful in detecting several FV3-like and un-typed ranaviruses in North America (see ^29^ for full pathogen screening protocol). After pathogen samples were collected, we rinsed animals with approximately 200 mL sterile distilled water and immediately rubbed a sterile cotton swab over the dorsal skin surface for later microbiome analysis (unpublished data). We then allowed individuals to sit in the same sterile water bath for 10 min to collect passive skin secretions. We gently tilted and swirled the container on each side to run water over the animal to capture secretions from all skin surfaces. We poured water/ skin secretion mixtures into aliquots within sterile 50 mL tubes, added 1 mL 6 M HCL, and immediately placed them on dry ice until transport to our laboratory for storage in a − 80 °C freezer.

### Peptide enrichment

To enrich skin secretions for small cationic peptides, we defrosted contents of a 50 mL aliquot and pumped slowly over an activated C-18 Sep-Pak filter (Waters Corp, Inc) following the protocol provided by^60^. We washed the Sep-Pak, then eluted 11 mL of final peptide mixture and removed 1 mL for immediate peptide quantification. The remaining 10 mL were concentrated to dryness by centrifugation under vacuum and stored at − 20 °C until use for growth inhibition assays (GIAs). We quantified peptides using a Micro BCA™ Protein Assay Kit (ThermoFisher #23235) compared to a bradykinin standard at concentrations of 0.5–200 µg/mL (Rollins-Smith et al.^60^). We resuspended dried peptides in HPLC-grade water to a concentration of either 2000 or 5000 µg/mL dependent on peptide amount recovered. We filter sterilized all resuspended peptides immediately before use in GIAs.

### Chytrid culture and zoospore harvest

For *Bd* GIAs we used a culture isolate provided by Joyce Longcore [JEL 197, isolated from a captive blue poison dart frog (*Dendrobates auratus*) in North America] at 21 °C and sub-cultured weekly to biweekly. Prior to planned zoospore harvest, we inoculated three sterile 1% tryptone agar plates infused with streptomycin and penicillin. After incubation at 21 °C for 3–5 days, we flooded each plate with 3 mL sterile 1% tryptone broth, immediately recollected the liquid, and pipetted over a 20 µM sterile filter attached to a flask and vacuum. We pipetted another 3 mL over each plate and let stand for 20 min, covered, before recollection. We flooded plates a third and final time with immediate collection over the vacuum filter. Based on hemocytometer counts, we diluted to stock mixture of 1 × 10^6^ zoospores/mL of recovered filtrate.

For *Bsal* GIAs*,* we used a culture isolate provided by Frank Pasmans and An Martel (AMFP13/1, isolated from a captive fire salamander in Europe). We harvested zoospores in a manner identical to *Bd* with the following exceptions: We maintained *Bsal* cultures and plates at 16 °C and used TGhL (1.6% tryptone, 0.4% gelatin hydrolysate and 0.2% lactose) broth for culture. We found *Bsal* growth to be slower than *Bd* and harvested zoospores between 5 and 7 days post agar plate inoculation. Strict biosecurity protocols were implemented when handling all live chytrid cultures.

### Growth inhibition assays (GIAs)

To quantify peptide in vitro activity, we performed GIAs of *Bd* zoospores of all samples. For a subset of samples with enough recovered peptides, we also performed GIAs against *Bsal*. Assays were performed in sterile 96-well microtiter plates as previously described^38^. Briefly, we pipetted 50 µL of zoospores (1 × 10^6^/mL; 500,000 total cells) mixed with 50 µL of serial peptide dilutions in HPLC water in triplicate to obtain final peptide concentrations of 25, 50, 100, 250, and 500 µg /mL in each well. To obtain more robust inhibition curves from individuals with enough recovered peptides, we tested additional concentrations of 1000 and 2500 µg/mL. Positive control wells contained 50 µL zoospores and 50 µL sterile HPLC water whereas negative control wells contained 50 µL of heat-killed zoospores (90 °C for 10 min) with 50 µL sterile HPLC water. We measured change in optical density at 490 nm between day 0 and 7 for *Bd* GIAs and day 0 and 9 for *Bsal* GIAs using a Biotek plate reader and Gen 5 2.01 software. We calculated % zoospore growth for each well based on change in absorbance compared to positive controls. We visually inspected all wells to confirm zoospore activity (or lack thereof) and for contamination. If a well had contamination, we removed it from our analysis and averaged two wells. If more than one well in a triplicate was contaminated, we re-ran a new assay for the entire plate. We averaged triplicate results for a single value of % zoospore growth at each peptide concentration for each sample to create zoospore growth inhibition curves and to use for statistical analysis.

### Statistical analysis

We performed generalized linear mixed models (GLMM) fit for a binomial response in RStudio^61^ via the lme4 package^62^ to test for effects of individual and field parameters on *Bd* zoospore growth at 500 µg/ mL skin peptide concentration. During data exploration, we observed a bimodal distribution and transformed % zoospore growth into a binomial response of weak (zoospore growth > 50%) or strong (zoospore growth < 50%) inhibition. We tested fixed effects of Region (ETN, MTN, AR), mass, *Bd* infection status (positive/negative), and toe lesion (presence/absence); we included year as a random effect. We did not include TL or SVL in our models because they were highly correlated with mass. We did not include sex as a predictor variable in GLMMs, as sexing hellbenders is unreliable either in immature individuals or in mature individuals outside of the breeding season without genetically testing^63^, which was not available for this study. We calculated z-scores for mass prior to analysis.

We evaluated relative model fit based on AIC_c_ of models from all possible combinations of fixed effects via the AICcmodavg package^64^ and considered models competitive when ΔAIC_c_ was < 2.0 (see Table 1). We model-averaged variables that appeared in one or more competitive models as recommended in^65^ to determine relative variable contribution in those models. Variables were considered significant if 95% C.I. for β calculated from model averaging did not cross 0.

We did not include ranavirus infection status in our models because of low number of positive individuals (n = 4), but since all positives were from the same site and sampling day, we reported changes in *Bd* zoospore growth between ranavirus- positive and ranavirus-negative individuals from that sampling event (n = 6).

To evaluate differences in anti-*Bd* vs anti-*Bsal* activity, we performed a paired samples Wilcoxon test for samples for which we performed both *Bd* and *Bsal* GIAs. Alpha was set at 0.05.

### MALDI-TOF mass spectrometry

We performed matrix-assisted laser-desorption ionization time-of-flight (MALDI-TOF) mass spectrometry on skin peptide samples from 19 individuals following the protocol from^66^. We prepared peptide samples at 1 mg/mL at a 1:1 v/v ratio of matrix (10 mg/mL α-cyano-4 hydroxycinnamic acid (Fluka, Sigma, St. Louis, MO, USA), 60% acetonitrile, 39.6% HPLC-grade water, and 0.4% trifluoroacetic acid (v/v/v)). We used an Ultraflex III time-of-flight mass spectrometer (Bruker Daltonics, Billerica, MA, USA) for delayed extraction, positive ion, reflector mode for collection of 250 laser shots per samples and analyzed and evaluated spectra from weights of 1000 to 4000 Da using Data Explorer v4.4 software. For each individual, we created a dataset of all molecular weights present with a relative intensity > 10% with corresponding isotope cluster areas. We grouped each peptide mixture into zoospore inhibition categories of 1–5 from weakest to strongest inhibitions with the following cutoffs: Category 1: 91–100% zoospore growth, Category 2: 76–90%, Category 3: 26–75%, 4: 11–25%, and Category 5: 0–10%. We summed isotope area across all samples to determine the most relatively abundant peptide mass signals. We performed indicator species analysis using package indicspecies^67^ in R Studio to determine peptide masses that best associated with each inhibition category.

## Data Availability

The datasets generated during and/or analyzed during the current study are available from the corresponding author on reasonable request.
